# *Sly* Represses Gene Expression in Sex Chromosomes

**DOI:** 10.1371/journal.pbio.1000243

**Published:** 2009-11-17

**Authors:** Caitlin Sedwick

**Affiliations:** Freelance Science Writer, San Diego, California, United States of America

The nuclei of eukaryotic cells can be thought of as a library containing the molecular “recipes,” or genes, for constructing the proteins and ribonucleic acid molecules used by a cell. Within the library, genes are grouped together into “books,” known as chromosomes, which consist of linear strands of DNA wrapped around specialized DNA-interacting proteins. Human body cells have 46 chromosomes. Of these, there are two copies of each of 22 unique chromosomes (one inherited from the mother and one from the father). The remaining two chromosomes are the sex chromosomes—two X chromosomes in a female and an X with a Y chromosome in a male. The Y chromosome is responsible for triggering the development of male physical characteristics. However, the functions of most Y-chromosomal genes are unknown. In this issue of *PLoS Biology*, Julie Cocquet, Paul Burgoyne, and colleagues provide new insights into the role of the mouse Y-chromosome gene *Sly*.

Until now, *Sly*'s role in cells has been a mystery, because unlike most genes, which have just one copy, *Sly* is present in multiple (over 100) copies. The conventional approach to studying a gene's function—knocking it out—involves replacing a normal gene with a disrupted version containing a selectable marker. This approach is unworkable with *Sly*, and other multicopy genes, because there are not enough selectable markers or man hours available to engineer that many disrupted copies. Therefore, the authors took an alternative approach: they designed transgenes to generate short hairpin RNAs (shRNAs) that could knock down SLY protein expression to undetectable levels and inserted these into mice. They found that the transgenic animals developed normally, but males were nearly sterile, with badly deformed sperm.

Sperm are generated via meiosis, a special form of cell division that involves two consecutive rounds of cell division without an intervening bout of chromosome duplication. In this way, four sperm cells are created from one original cell; each new cell contains one copy of every autosome and either the X or the Y chromosome.

As with regular cell division, during meiosis, all the chromosomes are packed up tightly for distribution among the new cells. Afterward, the new sperm's autosomes are unpacked for use. However, due to mechanisms that are poorly understood, transcription of most X- and Y-chromosome genes is maintained at a low level after meiosis. Multicopy genes, such as *Sly*, achieve a level of expression comparable to autosomal genes, perhaps because their extra copies make them more likely to be transcribed.

Until now, the only thing known about this repression mechanism was that it might be regulated by genes residing on the Y chromosome. Mice that have a large deletion in the long (q) arm of the Y chromosome (known as MSYq-deficient animals) show less repression. Interestingly, the deletion in MSYq-deficient animals covers the region where the *Sly* gene copies are found, and results in morphological sperm defects that are similar to those observed in *Sly*-shRNA sperm. These observations led the authors to test whether *Sly* might be involved in the repression of sex chromosome genes after meiosis.

Developing sperm cells are known to initially share their protein contents, and Cocquet et al. showed that SLY protein was indeed present in both X- and Y-chromosome–containing sperm in normal mice. Furthermore, consistent with the fact that SLY protein is predicted to have chromosome-binding activity, it was found near the sex chromosomes. This proximity led Cocquet et al. to examine whether loss of *Sly* might affect gene activity in sperm. In fact, they observed that many X- and Y-chromosome genes, and some autosomal genes, were more expressed (or de-repressed) in sperm from *Sly-*shRNA mice.

Taken together, the authors' data indicate that *Sly* plays a central role in the post-meiotic repression of sex chromosome genes. Paradoxically, this means *Sly* represses its own expression (along with that of other genes). This represents an important insight both into the repression mechanisms and the function of this heretofore uncharacterized gene.

**Cocquet J, Ellis PJI, Yamauchi Y, Mahadevaiah SK, Affara NA, et al (2009) The Multi-Copy Gene** ***Sly*** **Represses the Sex Chromosomes in the Male Mouse Germline after Meiosis. doi: 10.1371/journal.pbio. 1000244**

**Figure:**
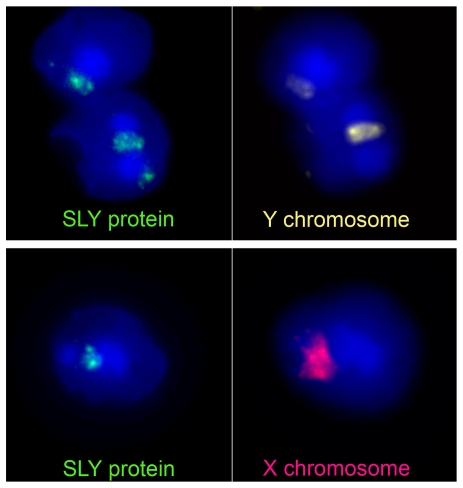
In male post-meiotic germ cells (that is, spermatids), SLY protein co-localizes with the X or the Y chromosome to repress transcription.

